# A Prediction Score Versus Procalcitonin and Presepsin for Predicting Bacteremia in the Emergency Department: A Single-Center Retrospective Cohort Study

**DOI:** 10.3390/diagnostics16152447

**Published:** 2026-08-03

**Authors:** Hirofumi Ohno, Sayumi Kato, Kenta Ishii, Kazuhiro Hiramatsu, Takeo Fukagawa

**Affiliations:** 1 Department of Emergency Medicine, Toyohashi Municipal Hospital, Toyohashi 441-8570, Japan; skla241008@yahoo.co.jp (S.K.); kenta-i@med.nagoya-u.ac.jp (K.I.); hiramatsu-kazuhiro@toyohashi-mh.jp (K.H.); 2 Department of Surgery, Teikyo University Hospital, Tokyo 173-8606, Japan; fukagawa.takeo.vn@teikyo-u.ac.jp

**Keywords:** bacteremia, prediction score, procalcitonin, presepsin, emergency department

## Abstract

**Background/Objectives**: Early prediction of bacteremia is clinically important in the emergency department (ED). We previously developed a prediction score using vital signs and routine blood tests. Whether procalcitonin (PCT) or presepsin (PSEP), biomarkers of bacterial infection, outperform this score for predicting bacteremia remains unclear. This study aimed to compare the predictive performance of the prediction score with PCT and PSEP for bacteremia in the ED. **Methods**: This single-center, retrospective cohort study included patients aged ≥16 years with suspected bacteremia in the ED between April 2020 and March 2021. The period was divided based on biomarker measured: Period 1 (PCT) and Period 2 (PSEP). The prediction score was compared with PCT within Period 1 and with PSEP within Period 2. Predictive performance for bacteremia was compared using the area under the receiver operating characteristic curve (AUC), and clinical utility was assessed by decision curve analysis (DCA). **Results**: Of 2922 patients (1539 in Period 1; 1383 in Period 2), the AUCs of the prediction score and PCT in Period 1 were 0.76 and 0.79 (*p* = 0.075). In Period 2, the score’s AUC (0.75) was significantly higher than PSEP’s (0.70, *p* = 0.013). DCA showed substantial net benefit for the score, with marginal incremental benefit from adding PCT or PSEP. **Conclusions**: A prediction score based on vital signs and routine blood tests showed predictive performance comparable to PCT and superior to PSEP for predicting bacteremia in the ED.

## 1. Introduction

Bacteremia is one of the most serious infectious conditions encountered in the emergency department (ED), associated with notable morbidity and mortality [1]. Early recognition is essential, as delays in the initiation of appropriate antibiotic therapy worsen outcomes [2,3]. Although blood culture is the diagnostic gold standard, it usually requires several days to yield results and is prone to false-negative and false-positive findings [4,5,6]. Therefore, various approaches have been developed to predict bacteremia at the time of ED presentation.

Prediction scores based on vital signs, laboratory data, and clinical findings are useful for the early identification of bacteremia [7,8]. We have previously developed and validated a prediction score composed of seven objective parameters derived from vital signs and routine blood tests, which demonstrated good predictive performance with an area under the receiver operating characteristic (ROC) curve (AUC) of 0.78 (95% confidence interval [CI]: 0.76–0.80) [9].

Furthermore, specific biomarkers of bacterial infections have received increasing attention as single-parameter predictors of bacteremia. Among these, procalcitonin (PCT) and presepsin (PSEP) are the most widely studied. PCT, a precursor of calcitonin, is induced in response to bacterial endotoxin and proinflammatory cytokines, with serum levels rising rapidly during systemic bacterial infection [10,11]. PSEP, a soluble fragment of CD14 released upon monocyte activation, reflects the severity of bacterial infection and has been reported to aid in the early diagnosis of sepsis and bacteremia [12,13].

Although both prediction scores and biomarkers demonstrate good predictive accuracy for bacteremia, to our knowledge, no study has directly compared the performance of a prediction score with that of these biomarkers. As a result, it remains unclear whether measuring a biomarker offers any advantage over information that is already available at presentation. This study compared the predictive ability of our prediction score with that of PCT and with that of PSEP for bacteremia in the ED setting.

## 2. Materials and Methods

### 2.1. Study Design and Setting

This single-center, retrospective cohort study was conducted in the ED of Toyohashi Municipal Hospital, a community hospital that serves as a tertiary emergency medical center in Aichi, Japan. This study adhered to the principles of the Declaration of Helsinki and was reported in accordance with the Transparent Reporting of a multivariable prediction model for Individual Prognosis Or Diagnosis (TRIPOD) statement. The study protocol was reviewed and approved by the Institutional Review Board of Toyohashi Municipal Hospital (approval number: 589; approval date: 3 May 2021), which waived the requirement for written informed consent owing to the retrospective use of anonymized medical record data.

### 2.2. Study Samples and Data Collection

We included patients aged ≥16 years who visited the ED during the study period, in whom bacteremia was suspected by the attending emergency physician, and from whom two or more blood cultures were collected. Suspicion of bacteremia was based on the clinical assessment by the attending emergency physician, without any specific criteria for patient selection. Patients with missing data were excluded from the primary analysis; sensitivity analyses using multiple imputation are described in Section 2.4. The present patients belonged to the validation cohort of our previous study [9] and did not overlap with the derivation cohort. The present analysis included patients who visited the ED between April 2020 and March 2021, when PCT and PSEP were measured. The study period was divided into Period 1 (April 2020–September 2020) for PCT and Period 2 (October 2020–March 2021) for PSEP, each constituting an independent cohort. During both periods, body temperature was measured, and routine blood tests and blood cultures were obtained upon ED admission. Data on age, sex, medical history, vital signs on arrival, routine blood test results, PCT, PSEP, blood culture results, final diagnosis, and outcomes were retrospectively collected from medical records. Blood cultures were obtained according to standard guidelines [14]. Briefly, after skin disinfection with alcohol and chlorhexidine, blood was drawn aseptically from two separate venipuncture sites, with at least 20 mL obtained per site. Each set was inoculated into one aerobic and one anaerobic bottle and processed on the BacT/ALERT^®^ 3D system (bioMérieux, Marcy l’Etoile, France). Sources of infection were classified based on the clinical course recorded in the medical records into the following categories: lung and pleura; urinary tract; biliary tract; gastrointestinal tract and peritoneum; skin and soft tissue; central nervous system; pelvis; head, eye, ear, nose, and trachea; bloodstream; others, including viral and fungal infections with no bloodstream involvement, infections of unknown source, and no infection. In cases with multiple sources of infection, overlap was recorded. Conditions without infection included heart failure, acute exacerbation of chronic obstructive pulmonary disease or interstitial pneumonia, acute pancreatitis, electrolyte disturbance, stroke, hyperglycemic crisis, heatstroke, and hypothermia.

### 2.3. Exposures and Outcomes

Exposures included the prediction score, PCT, and PSEP. The prediction score, which was previously developed and validated [9], consisted of seven parameters derived from vital signs and routine blood tests as follows: body temperature (BT), platelet count (Plt), neutrophil-to-lymphocyte ratio (NLR; calculated by dividing the neutrophil count by the lymphocyte count), albumin (Alb), bilirubin (Bil), creatinine (Cre), and lactate (Lac). Each variable was dichotomized according to predefined thresholds (BT ≥ 38.0 °C; Plt ≤ 100 × 10^9^/L; NLR ≥ 10; Alb ≤ 35 g/L; Bil ≥ 1.2 mg/dL; Cre ≥ 1.2 mg/dL; and Lac ≥ 18 mg/dL) and was assigned a value of 1 if the threshold was met and 0 otherwise. The score was then calculated as follows:

Prediction score = NLR × 3 + Plt × 2 + Bil × 2 + Lac × 2 + Cre + BT + Alb

In the original derivation study, eleven candidate variables were dichotomized according to previously established thresholds and entered into a multivariable logistic regression model with bacteremia as the dependent variable. Integer weights were derived from the regression coefficients, and variables were removed stepwise until discrimination no longer differed from that of the full model, yielding the present seven-variable score. In this study, the score was applied without refitting, using the previously published thresholds and weights.

PCT and PSEP were measured using the ARCHITECT^®^ i2000SR system (Abbott, Abbott Park, IL, USA) and the STACIA^®^ analyzer (LSI Medience, Tokyo, Japan), respectively. The primary outcome was bacteremia, defined as detection of bacterial growth in at least one blood culture bottle accompanied by initiation of antibiotic therapy directed against the isolated organism. Cases in which a positive culture did not require targeted antimicrobial treatment were classified as contaminated and counted as no-bacteremia. Organisms generally regarded as contaminants, including coagulase-negative staphylococci, *Corynebacterium* species, and *Bacillus* species, were treated as such unless the clinical context strongly suggested true bacteremia [15]. Conversely, organisms usually regarded as pathogenic were treated as contamination only when a definite non-infectious diagnosis fully accounted for the clinical presentation and no targeted antimicrobial treatment was given. Final classification as bacteremia or contamination was determined retrospectively by three of the authors, who each reviewed a separate subset of the cases based on the blood culture results and the corresponding clinical course in the medical records. Because all clinical and laboratory data, including biomarker results, were available at the time of review, the assessment was not blinded.

### 2.4. Statistical Analysis

Patient characteristics were described using medians with interquartile ranges (IQRs) for continuous variables and percentages for categorical variables. Continuous variables were compared using the Mann–Whitney U test, and categorical variables using the chi-square test. Using logistic regression analysis, ROC curves for bacteremia were generated, and the AUC was calculated for each predictor. The AUC of the prediction score was compared with that of PCT in Period 1 and with that of PSEP in Period 2, using the DeLong test. Calibration was assessed for logistic models fitted in each cohort, using calibration plots with loess smoothing in which patients were grouped into deciles of predicted risk, the calibration slope and intercept, the Brier score, and the scaled Brier score. The Hosmer–Lemeshow goodness-of-fit test was additionally performed. Optimism was estimated using bootstrap resampling with 1000 replications. To evaluate the clinical utility of the prediction score and the incremental value of adding PCT or PSEP, decision curve analysis (DCA) was performed using predicted probabilities from logistic regression models fitted in each cohort. Net benefit was calculated as:

$$NB=\frac{TP}{n}-\;\frac{FP}{n}\;\times \frac{Pt}{1-Pt}$$

where *TP* and *FP* denote the numbers of true and false positives, *n* the total number of patients, and *Pt* the threshold probability. Net benefit was compared with two reference strategies across a range of threshold probabilities: the “test-all” strategy, in which blood cultures are performed in all patients with suspected bacteremia, and the “test-none” strategy, in which blood cultures are performed in none and the net benefit is zero by definition. Confidence intervals were derived by bootstrap resampling, with models refitted within each resample. The 10% threshold was prespecified as the primary comparison point based on published guidance on blood culture indications [16], in which 10% was designated as the boundary between the “low” pretest probability category, where routine blood cultures are not recommended, and the “intermediate” category, where blood cultures are warranted. To our knowledge, this is the only explicitly proposed probability threshold for blood culture decision making in adult inpatients. Primary analyses were restricted to patients with complete biomarker and score data. Because biomarkers were measured at the discretion of the attending physician, we compared included and excluded patients using standardized mean differences (SMD), with an absolute value exceeding 0.10 indicating imbalance. As a sensitivity analysis, missing values were imputed using multiple imputation by chained equations (m = 30), with the outcome and all predictors included in the imputation model and biomarker concentrations log-transformed. Imputation was performed separately within each period because the biomarker measured differed between periods. AUCs were pooled using Rubin’s rules after logit transformation. We additionally performed a tipping-point analysis in which imputed biomarker values were shifted downward by up to 1.5 log units to assess robustness to departures from the missing-at-random assumption. Statistical significance was set at *p* < 0.05. All tests were two-tailed. All analyses were performed using R software version 4.0.3 (R Foundation for Statistical Computing, Vienna, Austria).

## 3. Results

### 3.1. Patient Flow

**Figure 1:**
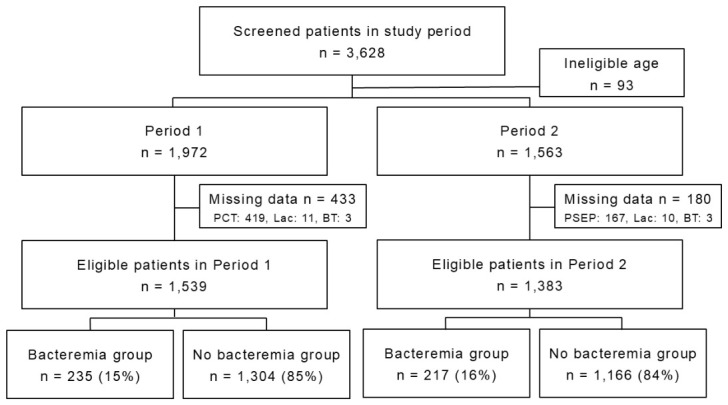
Study flowchart. Of the 3628 patients screened, 93 were excluded because of ineligible age. Of the remaining patients, 1972 were enrolled in Period 1 (April 2020–September 2020) and 1563 in Period 2 (October 2020–March 2021). After excluding patients with missing data (433 [PCT: 419, Lac: 11, BT: 3] and 180 [PSEP: 167, Lac: 10, BT: 3], respectively), 1539 and 1383 were included in the final analysis. Bacteremia was diagnosed in 235 (15%) and 217 (16%) patients in Periods 1 and 2, respectively. Abbreviations: BT, body temperature; Lac, lactate; PCT, procalcitonin; PSEP, presepsin.

During the study period, 3628 patients were initially eligible. After excluding 706 patients with missing data, 1539 patients in Period 1 and 1383 in Period 2 were included in the final analysis (Figure 1). Bacteremia was diagnosed in 235 patients (15%) in Period 1 and 217 patients (16%) in Period 2.

### 3.2. Patient Characteristics

Patient characteristics are summarized in Table 1. In the total cohort, the median age was 76 (IQR, 64–84) years, and 58% were male. Patients with bacteremia were significantly older, had higher rates of diabetes mellitus and solid tumor, were more likely to develop sepsis or septic shock, and more often required hospitalization or died, whereas male sex was more common in the no-bacteremia group. These differences were broadly consistent between Periods 1 and 2, with the following exceptions: in Period 1, the prevalence of diabetes mellitus and in-hospital mortality did not differ significantly between the two groups, whereas in Period 2, hematologic cancer was more common in the no-bacteremia group. In addition, body temperature, all laboratory parameters included in the prediction score, PCT, PSEP, and the prediction score itself showed significantly more abnormal values in the bacteremia group across all cohorts (Table 1).

### 3.3. Source of Infection

Sources of infection are also shown in Table 1. Urinary tract, biliary tract, and bloodstream sources were more common in the bacteremia group, whereas lung and pleura, others, and no infection were more common in the no-bacteremia group in the total cohort, as well as in both Period 1 and Period 2. Skin and soft tissue, and central nervous system sources were more frequent in the bacteremia group in the total cohort and in Period 2.

### 3.4. Causative Pathogens

The causative pathogens are shown in Table 2. Among the Gram-positive cocci, *Streptococcus* species and *Staphylococcus aureus* were the most common, whereas Enterobacteriaceae, such as *Escherichia coli* and *Klebsiella* species, predominated among the Gram-negative rods. Of the 41 contamination cases, 14 involved organisms that are usually regarded as true pathogens [17]: *Staphylococcus aureus* (*n* = 3), *Escherichia coli* (*n* = 3), *Klebsiella* species (*n* = 4), *Enterococcus* species (*n* = 2), other Enterobacteriaceae (*n* = 1), and *Candida* species (*n* = 1). In 10 of these, the organism was recovered from only one of the two sets, and in all 14 a definite non-infectious diagnosis accounted for the presentation and no directed antimicrobial therapy was administered.

### 3.5. Predictive Performance of the Prediction Score, PCT, and PSEP

**Figure 2:**
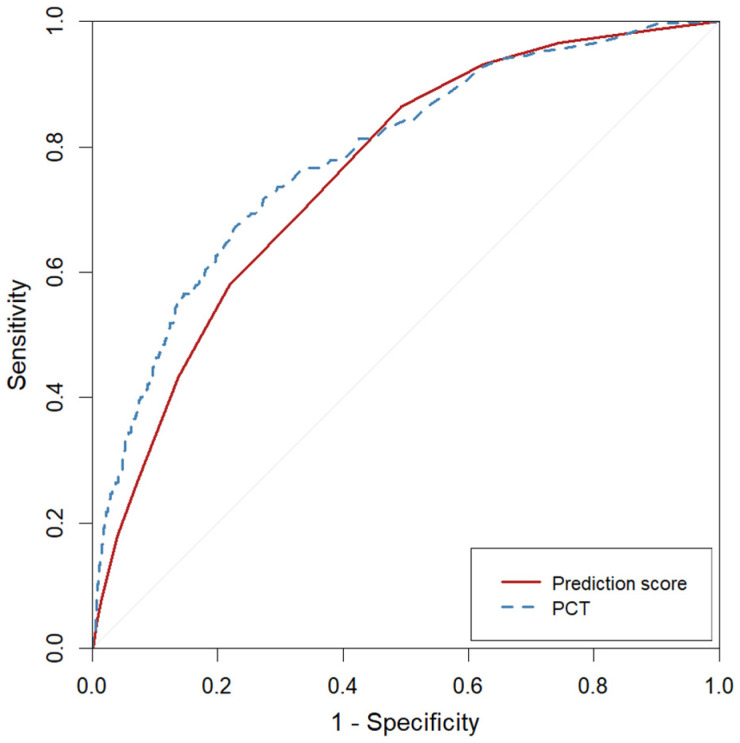
Receiver operating characteristic curves of the prediction score and procalcitonin for predicting bacteremia in Period 1. Analyses included 1539 patients, of whom 235 (15%) had bacteremia. Solid red line: prediction score; dashed blue line: procalcitonin. Curves were derived from the prediction score as a continuous variable and from procalcitonin concentrations as a continuous variable. The area under the curve was 0.76 (95% CI, 0.72–0.79) for the prediction score and 0.79 (95% CI, 0.75–0.82) for procalcitonin, with a difference of 0.030 (95% CI, −0.003 to 0.062; *p* = 0.075, DeLong test).

**Figure 3:**
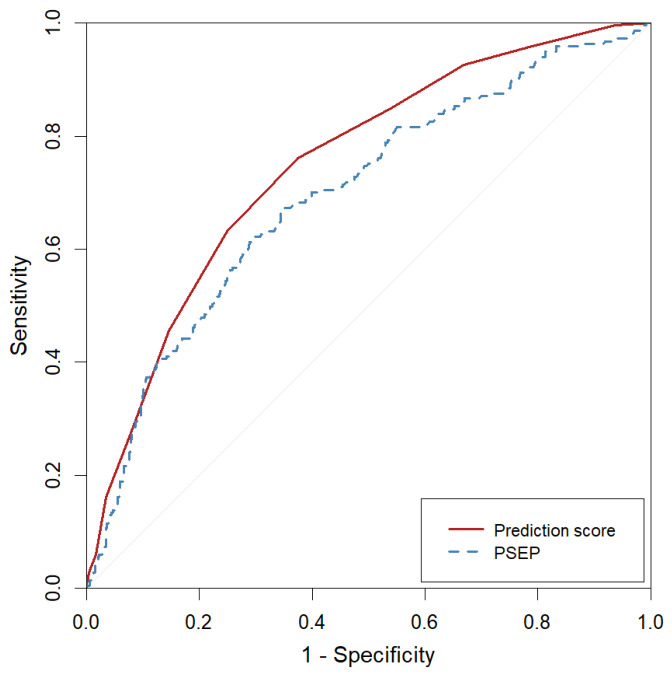
Receiver operating characteristic curves of the prediction score and presepsin for predicting bacteremia in Period 2. Analyses included 1383 patients, of whom 217 (16%) had bacteremia. Solid red line: prediction score; dashed blue line: presepsin. Curves were derived from the prediction score as a continuous variable and from presepsin concentrations as a continuous variable. The area under the curve was 0.75 (95% CI, 0.71–0.78) for the prediction score and 0.70 (95% CI, 0.66–0.73) for presepsin, with a difference of 0.053 (95% CI, 0.011 to 0.095; *p* = 0.013, DeLong test).

In Period 1, the AUCs of the prediction score and PCT were 0.76 (95% CI, 0.72–0.79) and 0.79 (95% CI, 0.75–0.82), respectively, with no significant difference between the two (*p* = 0.075; Figure 2). In Period 2, the AUCs of the prediction score and PSEP were 0.75 (95% CI, 0.71–0.78) and 0.70 (95% CI, 0.66–0.73), respectively. The AUC of the prediction score was significantly higher than that of PSEP (*p* = 0.013; Figure 3).

### 3.6. Calibration

In Period 1, calibration plots (Figure S1) demonstrated close agreement between predicted probabilities and observed proportions of bacteremia across the entire range of predicted risk for both the prediction score and PCT, with predicted probabilities falling within the 95% confidence intervals of the observed proportions in all deciles. Optimism estimated by bootstrap resampling with 1000 replications was negligible for both measures, indicating no meaningful overfitting, and the Hosmer–Lemeshow test showed no evidence of poor fit for either the prediction score or PCT. In Period 2, calibration plots (Figure S2) similarly demonstrated close agreement between predicted probabilities and observed proportions for the prediction score, with predicted probabilities falling within the 95% confidence intervals of the observed proportions in all deciles. For PSEP, agreement was slightly less consistent, with the predicted probability lying marginally above the 95% confidence interval of the observed proportion in one decile. Optimism was again negligible for both measures, and the Hosmer–Lemeshow test showed no evidence of poor fit. Calibration and overall performance measures for both periods are summarized in Table S1.

### 3.7. Decision Curve Analysis

**Figure 4:**
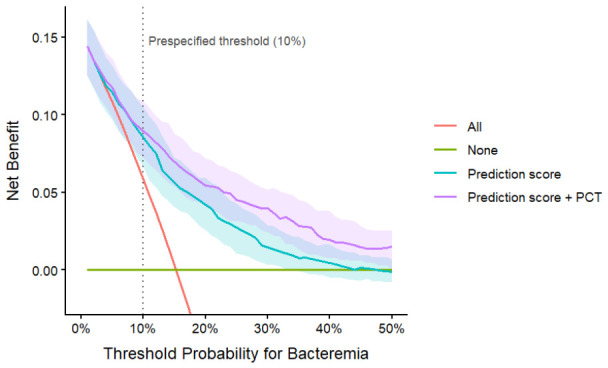
Decision curve analysis of prediction score with and without procalcitonin (Period 1). The *x*-axis indicates “Threshold Probability for Bacteremia”; the *y*-axis indicates “Net Benefit”. Two reference strategies are shown for comparison: the “All” strategy (red line), in which blood cultures are obtained in every patient with suspected bacteremia regardless of predicted risk, and the “None” strategy (olive line), in which blood cultures are obtained in no patient and the net benefit is therefore zero by definition. A strategy is clinically useful only if its curve lies above both reference lines at the threshold probability of interest. The dotted vertical line indicates the prespecified threshold probability of 10%. Shaded areas represent 95% confidence intervals derived from bootstrap resampling. The prediction score (cyan line) provided a higher net benefit than the test-all strategy across a wide range of threshold probabilities. Adding procalcitonin to the prediction score (purple line) yielded only a marginal incremental benefit. Abbreviation: PCT, procalcitonin.

**Figure 5:**
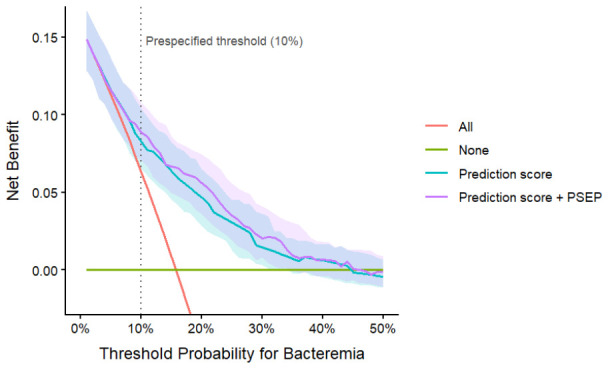
Decision curve analysis of prediction score with and without presepsin (Period 2). The *x*-axis indicates “Threshold Probability for Bacteremia”; the *y*-axis indicates “Net Benefit”. Two reference strategies are shown for comparison: the “All” strategy (red line), in which blood cultures are obtained in every patient with suspected bacteremia regardless of predicted risk, and the “None” strategy (olive line), in which blood cultures are obtained in no patient and the net benefit is therefore zero by definition. A strategy is clinically useful only if its curve lies above both reference lines at the threshold probability of interest. The dotted vertical line indicates the prespecified threshold probability of 10%. Shaded areas represent 95% confidence intervals derived from bootstrap resampling. The prediction score (cyan line) provided a higher net benefit than the test-all strategy across a wide range of threshold probabilities. Adding presepsin to the prediction score (purple line) yielded only a marginal incremental benefit. Abbreviation: PSEP, presepsin.

The clinical utility of the prediction score alone and in combination with PCT or PSEP was assessed using DCA. Across a wide range of threshold probabilities, the prediction score provided a higher net benefit than both the “test-all” and “test-none” strategies in Period 1 (Figure 4) and Period 2 (Figure 5). At the prespecified 10% threshold, the net benefit of the prediction score was 0.086 (95% CI, 0.068–0.104) in Period 1 and 0.083 (95% CI, 0.065–0.103) in Period 2, compared with 0.059 (95% CI, 0.039–0.080) and 0.063 (95% CI, 0.042–0.085) for the test-all strategy, respectively. The incremental net benefit obtained by adding PCT to the prediction score in Period 1 was 0.004 (95% CI, −0.004 to 0.011), and that obtained by adding PSEP in Period 2 was 0.006 (95% CI, −0.001 to 0.013).

### 3.8. Missing Data and Sensitivity Analysis

In Period 1, 433 of 1972 patients (22.0%) were excluded from the primary analysis owing to missing PCT (*n* = 419) or score components (*n* = 14); in Period 2, 180 of 1563 patients (11.5%) were excluded owing to missing PSEP (*n* = 167) or score components (*n* = 13), described in Table S2. In Period 1, excluded patients had lower body temperature, lower NLR, higher albumin, lower prediction scores, and a lower prevalence of bacteremia and sepsis than included patients, whereas in-hospital mortality did not differ. In Period 2, the two groups were comparable across all characteristics examined except for a lower prevalence of bacteremia among excluded patients (Table S3). After multiple imputation of the full cohorts, the AUCs were similar to the complete-case estimates and the direction of the between-group differences was unchanged. In Period 1, the AUC was 0.78 (95% CI 0.75–0.82) for PCT and 0.76 (0.73–0.78) for the prediction score, with a difference of 0.03 (−0.01 to 0.06; *p* = 0.12). In Period 2, the AUC was 0.69 (0.65–0.73) for PSEP and 0.74 (0.71–0.77) for the prediction score, with a difference of −0.05 (−0.09 to −0.003; *p* = 0.04). Tipping-point analyses shifting imputed biomarker values downward by up to 1.5 log units yielded AUCs of 0.78 to 0.79 in Period 1 and 0.69 to 0.70 in Period 2, without altering the conclusions of either period.

## 4. Discussion

In this retrospective cohort study of 2922 ED patients with suspected bacteremia, we compared the predictive performance of our previously developed prediction score with that of PCT and PSEP. In Period 1, the prediction score and PCT showed comparable discriminative ability for bacteremia, with no significant difference in AUC. In Period 2, the prediction score showed significantly greater discriminative ability than PSEP, with a significantly higher AUC. The prediction score also showed good calibration, with predicted probabilities falling within the 95% confidence intervals of the observed proportions across all deciles of predicted risk. In this cohort, the prediction score, based on vital signs and routine blood tests, showed predictive performance comparable to PCT and higher than PSEP for detecting bacteremia in the ED.

We further assessed clinical utility using DCA. At the prespecified 10% threshold, the score exceeded the net benefit of the test-all strategy by 0.027 in Period 1 and 0.020 in Period 2, corresponding to approximately 24 and 18 fewer unnecessary blood cultures per 100 patients without missing any additional case of bacteremia. Adding PCT (0.004) or PSEP (0.006) yielded fewer than one additional true bacteremia case detected per 100 patients. These findings suggest that, in this cohort, the prediction score may help guide blood culture use, whereas adding either biomarker appeared to offer little further benefit.

PCT and PSEP have been increasingly studied as single-parameter predictors of bacterial infection and bacteremia, with previously reported AUCs of 0.72–0.88 for PCT and 0.60–0.79 for PSEP in predicting bacteremia [18]. Our results were consistent with this range, with AUCs of 0.79 for PCT and 0.70 for PSEP. However, to the best of our knowledge, no previous study has directly compared the performance of a prediction score based on routinely available parameters with those of these biomarkers in the ED. The present study therefore provides evidence that a simple score-based approach may achieve predictive accuracy comparable to that of these biomarkers in this setting.

These results can be explained by two mechanisms. First, bacteremia frequently leads to organ dysfunction, and these downstream consequences might be reflected more comprehensively by the prediction score rather than by single biomarkers [19,20]. In our study, the bacteremia group had a significantly higher prevalence of sepsis and septic shock, both of which represent infection-induced organ dysfunction. Consistent with this, all parameters constituting the prediction score, including those representing coagulation (Plt), acute-phase response (Alb), hepatic function (Bil), renal function (Cre), and tissue perfusion (Lac), were significantly more abnormal in the bacteremia group across all cohorts. The prediction score thus appears to integrate multiple aspects of the host response to infection, including both inflammatory and organ-dysfunction components, whereas PCT and PSEP primarily reflect the inflammatory response to bacterial infection [11,13]. This multifaceted nature may contribute to its comparable or superior performance compared with single-parameter biomarkers, particularly in ED patients with heterogeneous clinical presentations. Second, the predictive performance of PCT and PSEP may have been influenced by biomarker kinetics. PCT levels become detectable within 2–4 h of bacterial infection and peak at 24 h [10], whereas PSEP increases more rapidly, peaking at approximately 3 h, but with a markedly short half-life of 4–5 h [21]. Accordingly, in the ED, where blood is frequently drawn during the early course of infection, PCT may not have reached diagnostic concentrations, while PSEP may have already declined from its peak. In contrast, parameters constituting the prediction score change rapidly in response to infection and persist throughout the clinical course. In particular, NLR increases within hours of infection onset and remains elevated for at least several days [22,23]. Similarly, serum lactate remains elevated until the underlying infection and hemodynamic compromise are adequately treated [19]. These characteristics suggest that the prediction score parameters may more accurately reflect pathophysiological changes at ED presentation than biomarkers with either delayed onset or short half-lives.

These findings may have clinical implications, although they remain hypothesis-generating. If the predictive performance observed here is confirmed in other settings, routine measurement of these biomarkers in the ED may not always be necessary. This would reduce not only the direct financial costs of biomarker assays but also the indirect workload on clinical laboratory technologists. In addition, because the parameters constituting the score are already obtained as part of standard ED evaluation, the score is available without any additional measurement time. Moreover, while PCT and PSEP are not universally available, particularly in community hospitals or during off-hours, the score can be applied in virtually any clinical setting, allowing a uniform approach to risk assessment regardless of institutional resources. Although these considerations are derived from the present single-center cohort and warrant confirmation in further studies, the score may therefore serve as a primary tool for early risk stratification of ED patients with suspected bacteremia.

This study has several limitations. First, it was a single-center retrospective study, which may limit generalizability of the findings. The present patients corresponded to the validation cohort of our previous study, in which the score was developed; although they did not overlap with the derivation cohort, the present analysis therefore does not constitute independent external validation. Second, PCT and PSEP were measured in different periods. A direct comparison of the two biomarkers was not an objective of this study, and each comparison was performed within a single cohort; however, their relative performance cannot be inferred from our data. Moreover, as the study period overlapped with the COVID-19 pandemic, differences in case mix, infection patterns, and pandemic-era practice may have affected comparisons between periods [24]. Third, the decision to obtain blood cultures was based on the clinical judgment of the attending emergency physician without standardized criteria, which may have introduced selection bias. However, no universally accepted criteria for initiating blood cultures currently exist, and the decision is generally left to clinical discretion in most ED settings. In addition, each case was classified by one of three reviewing authors, with each author reviewing a separate subset of cases; classification was performed without formal blinding or duplicate assessment, and interobserver agreement was therefore not evaluated. Consequently, interobserver variability and incorporation bias cannot be entirely excluded. Moreover, a small number of isolates of organisms usually regarded as pathogenic were classified as contamination on clinical grounds, and residual misclassification cannot be excluded. Fourth, a considerable number of patients were excluded from the primary analysis owing to missing data, mainly unmeasured biomarkers. Two mechanisms may account for this. First, PCT and PSEP are not routinely ordered in daily practice, and attending physicians may simply have failed to request them; consistent with this, missing values were more frequent for PCT, which was collected during the earlier phase of the study, than for PSEP. Second, selection bias may have operated whereby biomarkers and other laboratory tests were less likely to be ordered in patients with lower illness severity; consistent with this, excluded patients had a lower prevalence of bacteremia than included patients in both periods. However, sensitivity analyses using multiple imputation of the full cohorts yielded results consistent with the complete-case analysis in both direction and statistical significance, and a tipping-point analysis assuming that unmeasured biomarker concentrations were systematically lower did not alter the conclusions. Fifth, the predictive performance of combinations of the prediction score with PCT or PSEP was not evaluated in detail, as the primary aim was to compare the score with these biomarkers rather than to develop a combined model. Instead, DCA was employed to assess the incremental clinical utility of adding PCT or PSEP to the score, which consistently demonstrated only marginal benefit. Similarly, subgroup analyses were not prespecified and are not presented; whether the performance of the score differs according to the source of infection, patient age, or renal function warrants investigation in larger studies. Finally, this study evaluated predictive performance only; blood culture utilization, antibiotic prescribing, patient outcomes, and cost-effectiveness were not assessed, and the potential clinical and economic benefits suggested here therefore remain hypothetical. To address these limitations, external validation and prospective studies in other institutions are needed to evaluate the performance of the score and these biomarkers, as well as its impact on clinical practice.

## 5. Conclusions

A prediction score based on vital signs and routine blood tests showed predictive performance comparable to PCT and superior to PSEP for predicting bacteremia in the ED. The score may offer a practical approach to the early identification of bacteremia in the ED, although further studies are required before clinical implementation.

## Figures and Tables

**Table 1:** Characteristics of included patients.

Total (*n* = 2922)				
	Total	Bacteremia Group (*n* = 452 [15%])	No-Bacteremia Group (*n* = 2470 [85%])	*p* Value
Variables				
Basic characteristics				
Age (years)	76 (64, 84)	77 (70, 85)	76 (62, 84)	<0.001
Male sex	1689 (58)	235 (52)	1454 (59)	<0.01
Comorbidities				
Heart failure	198 (7)	30 (7)	168 (7)	0.98
Chronic lung disease	207 (7)	23 (5)	184 (7)	0.09
Liver cirrhosis	52 (2)	11 (2)	41 (2)	0.34
Chronic renal disease	196 (7)	35 (8)	161 (7)	0.39
Diabetes mellitus	599 (21)	119 (26)	480 (19)	0.001
Autoimmune disease	135 (5)	28 (6)	107 (4)	0.11
Solid tumor	724 (25)	140 (31)	584 (24)	0.001
Hematologic cancer	120 (4)	13 (3)	107 (4)	0.19
Outcomes				
Hospitalization	2259 (77)	429 (95)	1830 (74)	<0.001
In-hospital mortality	260 (9)	60 (13)	200 (8)	<0.001
Vital signs				
Body temperature (°C)	37.7 (37.0, 38.5)	38.2 (37.4, 39.1)	37.7 (36.9, 38.4)	<0.001
Laboratory data				
Platelet count (×10^9^/L)	204 (150, 266)	169 (125, 220)	211 (157, 273)	<0.001
Neutrophil lymphocyte ratio	9.4 (5.2, 17.5)	16.7 (9.4, 30.9)	8.6 (4.8, 15.5)	<0.001
Albumin (g/L)	33 (29, 38)	31 (27, 36)	34 (29, 38)	<0.001
Bilirubin (mg/dL)	0.8 (0.5, 1.1)	1.0 (0.7, 1.5)	0.7 (0.5, 1.1)	<0.001
Creatinine (mg/dL)	0.9 (0.7, 1.2)	1.1 (0.8, 1.7)	0.8 (0.7, 1.2)	<0.001
Lactate (mg/dL)	14 (10, 21)	19 (12, 31)	13 (10, 20)	<0.001
Procalcitonin (ng/mL)	n/a	n/a	n/a	n/a
Presepsin (pg/mL)	n/a	n/a	n/a	n/a
Prediction score	4 (2, 6)	6 (4, 8)	4 (2, 5)	<0.001
Source of infection				
Lung and pleura	705 (24)	48 (11)	657 (27)	<0.001
Urinary tract	488 (17)	175 (39)	313 (13)	<0.001
Biliary tract	212 (7)	73 (16)	139 (6)	<0.001
Gastrointestinal tract and peritoneum	195 (6)	27 (6)	168 (7)	0.56
Skin and soft tissue	173 (6)	39 (9)	134 (5)	0.01
Bloodstream	62 (2)	60 (13)	2 (0.1)	<0.001
Central nervous system	8 (0.3)	4 (0.9)	4 (0.2)	0.03
Pelvis	21 (0.7)	4 (0.9)	17 (0.7)	0.88
Head, eye, ear, nose, and trachea	41 (1)	3 (0.7)	38 (2)	0.22
Others	416 (14)	22 (5)	394 (16)	<0.001
No infection	615 (21)	0 (0)	615 (25)	<0.001
Patient condition				
Sepsis	372 (13)	197 (44)	175 (7)	<0.001
Septic shock	79 (3)	47 (10)	32 (1)	<0.001
**Period 1 (*n* = 1539)**				
	**Total**	**Bacteremia Group** **(*n* = 235 [15%])**	**No-Bacteremia Group** **(*n* = 1304 [85%])**	***p* Value**
Variables				
Basic characteristics				
Age (years)	75 (63, 84)	76 (70, 84)	75 (61, 84)	<0.01
Male sex	879 (57)	122 (52)	757 (58)	<0.01
Comorbidities				
Heart failure	89 (6)	13 (6)	76 (6)	0.86
Chronic lung disease	87 (6)	11 (5)	76 (6)	0.48
Liver cirrhosis	23 (2)	4 (2)	19 (2)	0.77
Chronic renal disease	88 (6)	18 (8)	70 (5)	0.16
Diabetes mellitus	289 (19)	50 (21)	239 (18)	0.29
Autoimmune disease	71 (5)	14 (6)	57 (4)	0.73
Solid tumor	375 (24)	72 (31)	303 (23)	0.02
Hematologic cancer	60 (4)	9 (4)	51 (4)	0.29
Outcomes				
Hospitalization	1149 (75)	219 (93)	930 (71)	<0.001
In-hospital mortality	111 (7)	22 (9)	89 (7)	0.17
Vital signs				
Body temperature (°C)	37.8 (37.0, 38.6)	38.4 (37.5, 39.2)	37.7 (37.0, 38.5)	<0.001
Laboratory data				
Platelet count (×10^9^/L)	204 (151, 264)	167 (119, 220)	211 (157, 271)	<0.001
Neutrophil lymphocyte ratio	9.1 (5.1, 17.1)	16.8 (11.9, 22.0)	8.2 (4.7, 15.2)	<0.001
Albumin (g/L)	34 (30, 39)	33 (28, 37)	35 (30, 39)	<0.001
Bilirubin (mg/dL)	0.8 (0.6, 1.2)	1.0 (0.7, 1.6)	0.7 (0.5, 1.1)	<0.001
Creatinine (mg/dL)	0.8 (0.7, 1.2)	1.1 (0.8, 1.5)	0.8 (0.5, 1.1)	<0.001
Lactate (mg/dL)	13 (10, 20)	19 (12, 30)	13 (9, 18)	<0.001
Procalcitonin (ng/mL)	0.2 (0.1, 0.8)	1.9 (0.3, 14.0)	0.1 (0.1, 0.5)	<0.001
Presepsin (pg/mL)	n/a	n/a	n/a	n/a
Prediction score	4 (2, 6)	6 (4, 8)	3 (1, 5)	<0.001
Source of infection				
Lung and pleura	344 (22)	20 (9)	324 (25)	<0.001
Urinary tract	265 (17)	96 (41)	169 (13)	<0.001
Biliary tract	120 (8)	45 (19)	75 (6)	<0.001
Gastrointestinal tract and peritoneum	109 (7)	19 (8)	90 (7)	0.52
Skin and soft tissue	105 (7)	16 (7)	89 (7)	>0.99
Bloodstream	21 (1)	19 (8)	2 (0.2)	<0.001
Central nervous system	4 (0.3)	1 (0.4)	3 (0.2)	0.48
Pelvis	9 (0.6)	1 (0.4)	8 (0.6)	>0.99
Head, eye, ear, nose, and trachea	25 (2)	1 (0.4)	24 (2)	0.16
Others	237 (15)	17 (7)	220 (17)	<0.001
No infection	307 (20)	0 (0)	307 (24)	<0.001
Patient condition				
Sepsis	186 (12)	97 (41)	89 (7)	<0.001
Septic shock	34 (2)	19 (8)	15 (1)	<0.001
**Period 2 (*n* = 1383)**				
	**Total**	**Bacteremia Group** **(*n* = 217 [16%])**	**No-Bacteremia Group** **(*n* = 1166 [84%])**	***p* Value**
Variables				
Basic characteristics				
Age (years)	77 (67, 85)	79 (70, 86)	77 (65, 85)	0.02
Male sex	810 (59)	113 (52)	697 (60)	0.03
Comorbidities				
Heart failure	109 (8)	17 (8)	92 (8)	0.98
Chronic lung disease	120 (9)	12 (6)	108 (9)	0.07
Liver cirrhosis	29 (2)	7 (3)	22 (2)	0.20
Chronic renal disease	108 (8)	17 (8)	91 (8)	0.99
Diabetes mellitus	310 (22)	69 (32)	241 (21)	<0.001
Autoimmune disease	64 (5)	14 (7)	50 (4)	0.16
Solid tumor	349 (25)	68 (31)	281 (24)	0.02
Hematologic cancer	60 (4)	4 (2)	56 (5)	0.049
Outcomes				
Hospitalization	1110 (80)	210 (97)	900 (77)	<0.001
In-hospital mortality	149 (11)	38 (18)	111 (10)	<0.001
Vital signs				
Body temperature (°C)	37.6 (36.9, 38.4)	38.1 (37.1, 39.1)	37.6 (36.8, 38.3)	<0.001
Laboratory data				
Platelet count (×10^9^/L)	203 (150, 268)	170 (129, 224)	211 (158, 277)	<0.001
Neutrophil lymphocyte ratio	9.8 (5.3, 18.3)	16.7 (9.7, 31.0)	9.0 (5.0, 16.2)	<0.001
Albumin (g/L)	32 (28, 37)	30 (26, 34)	33 (29, 38)	<0.001
Bilirubin (mg/dL)	0.7 (0.5, 1.1)	0.9 (0.7, 1.3)	0.7 (0.5, 1.1)	<0.001
Creatinine (mg/dL)	0.9 (0.7, 1.3)	1.1 (0.8, 1.8)	0.9 (0.7, 1.2)	<0.001
Lactate (mg/dL)	14 (10, 21)	19 (13, 31)	14 (10, 21)	<0.001
Procalcitonin (ng/mL)	n/a	n/a	n/a	n/a
Presepsin (pg/mL)	400 (240, 840)	770 (370, 1860)	370 (230, 670)	<0.001
Prediction score	4 (2, 6)	6 (5, 8)	4 (2, 5)	<0.001
Source of infection				
Lung and pleura	361 (26)	28 (13)	333 (29)	<0.001
Urinary tract	223 (16)	79 (36)	144 (12)	<0.001
Biliary tract	92 (7)	28 (13)	64 (6)	<0.01
Gastrointestinal tract and peritoneum	86 (6)	8 (4)	78 (7)	0.09
Skin and soft tissue	68 (5)	23 (11)	45 (4)	<0.01
Bloodstream	41 (3)	41 (19)	0 (0)	<0.001
Central nervous system	4 (0.3)	3 (1)	1 (<0.1)	0.01
Pelvis	12 (1)	3 (1)	9 (1)	0.42
Head, eye, ear, nose, and trachea	16 (1)	2 (1)	14 (1)	>0.99
Others	179 (13)	5 (2)	174 (15)	<0.001
No infection	308 (22)	0 (0)	308 (26)	<0.001
Patient condition				
Sepsis	186 (13)	100 (46)	86 (7)	<0.001
Septic shock	45 (3)	28 (13)	17 (2)	<0.001

Patient characteristics were analyzed using the Mann–Whitney U test for continuous variables, which were described as medians with interquartile ranges, and the chi-square test for categorical variables, which were presented as numbers (%). Lactate values are presented in mg/dL; the threshold of 18 mg/dL used in the prediction score corresponds to approximately 2.0 mmol/L.

**Table 2:** Causative organisms in the study cohorts.

	Total (*n* = 2922)	Period 1 (*n* = 1539)	Period 2 (*n* = 1383)
Bacteremia, *n* (%)	452 (15)	235 (15)	217 (16)
Gram-positive cocci			
*Staphylococcus aureus*	47 (10)	15 (6)	32 (15)
Coagulase-negative staphylococci	10 (2)	7 (3)	3 (1)
*Streptococcus* species	65 (14)	37 (16)	28 (13)
*Enterococcus* species	17 (4)	9 (4)	8 (4)
Gram-negative rods			
*Escherichia coli*	165 (37)	89 (38)	76 (35)
*Klebsiella* species	72 (16)	47 (20)	25 (12)
Other Enterobacteriaceae	31 (7)	14 (6)	17 (8)
*Pseudomonas aeruginosa*	11 (2)	6 (3)	5 (2)
Anaerobes	31 (7)	18 (8)	13 (6)
Other organisms	26 (6)	16 (6)	10 (6)
Contamination, *n* (%)	41 (1)	25 (2)	16 (1)
Viridans streptococci	12 (30)	8 (32)	4 (25)
Coagulase-negative staphylococci	11 (27)	5 (20)	6 (38)
*Staphylococcus aureus*	3 (7)	2 (8)	1 (6)
*Enterococcus* species	2 (5)	2 (8)	0 (0)
*Escherichia coli*	3 (7)	2 (8)	1 (6)
*Klebsiella* species	4 (10)	2 (8)	2 (13)
Other Enterobacteriaceae	1 (2)	1 (4)	0 (0)
*Bacillus* species	1 (2)	1 (4)	0 (0)
Anaerobes	2 (5)	1 (4)	1 (6)
*Candida* species	1 (2)	1 (4)	0 (0)
*Corynebacterium* species	1 (2)	0 (0)	1 (6)

Data are presented as *n* (%). Percentages for bacteremia and contamination are calculated using the total number of patients as the denominator; percentages for individual organisms are calculated using the number of bacteremia or contamination episodes, respectively, as the denominator. Polymicrobial episodes were counted once as a bacteremia event, but each isolated organism is listed separately; therefore, the sum of organisms exceeds the number of episodes. Organisms usually considered true pathogens (*S. aureus*, *Enterococcus* species, *Escherichia coli*, *Klebsiella* species, other Enterobacteriaceae, and *Candida* species; *n* = 14) were classified as contamination by the reviewing authors because a definite non-infectious diagnosis fully accounted for the clinical presentation and no targeted antimicrobial therapy was administered.

## Data Availability

The datasets generated and analyzed in the current study are available from the corresponding author upon reasonable request.

## Supplementary Materials

The following supporting information can be downloaded at: https://www.mdpi.com/article/10.3390/diagnostics16152447/s1, Figure S1: Calibration plots of the prediction score and procalcitonin (Period 1). Figure S2: Calibration plots of the prediction score and presepsin (Period 2). Table S1: Calibration and overall performance of the prediction score and biomarkers in each period. Table S2: Patient screening, exclusion, and inclusion in each period. Table S3: Comparison of baseline characteristics, biomarkers, and outcomes between included and excluded patients in each period.

## Abbreviations

The following abbreviations are used in this manuscript:

Alb	Albumin
AUC	Area under the receiver operating characteristic curve
Bil	Bilirubin
BT	Body temperature
CI	Confidence interval
Cre	Creatinine
DCA	Decision curve analysis
ED	Emergency department
IQR	Interquartile range
Lac	Lactate
NLR	Neutrophil to lymphocyte ratio
PCT	Procalcitonin
Plt	Platelet count
PSEP	Presepsin
ROC	Receiver operating characteristic
SMD	Standardized Mean Difference
TRIPOD	Transparent Reporting of a multivariable prediction model for Individual Prognosis Or Diagnosis

## References

[1] Chiang H.-Y., Chen T.-C., Lin C.-C., Ho L.-C., Kuo C.-C., Chi C.-Y. (2021). Trend and Predictors of Short-Term Mortality of Adult Bacteremia at Emergency Departments: A 14-Year Cohort Study of 14 625 Patients. Open Forum Infect. Dis..

[2] De la Rosa-Riestra S., Martínez Pérez-Crespo P.M., Pérez Rodríguez M.T., Sousa A., Goikoetxea J., Reguera Iglesias J.M., Armiñanzas C., López-Hernández I., López-Cortés L.E., Rodríguez-Baño J. (2024). Mortality Impact of Further Delays in Active Targeted Antibiotic Therapy in Bacteraemic Patients That Did Not Receive Initial Active Empiric Treatment: Results from the Prospective, Multicentre Cohort PROBAC. Int. J. Infect. Dis..

[3] Van Heuverswyn J., Valik J.K., Desirée van der Werff S., Hedberg P., Giske C., Nauclér P. (2023). Association between Time to Appropriate Antimicrobial Treatment and 30-Day Mortality in Patients with Bloodstream Infections: A Retrospective Cohort Study. Clin. Infect. Dis..

[4] Opota O., Croxatto A., Prod’hom G., Greub G. (2015). Blood Culture-Based Diagnosis of Bacteraemia: State of the Art. Clin. Microbiol. Infect..

[5] Hsu P.-H., Chang R., Yin C.-H., Chen Y.-S., Chen J.-S. (2024). Association between Blood Culture Turnaround Time and Clinical Prognosis in Emergency Department Patients with Community Acquired Bloodstream Infection: A Retrospective Study Based on Electronic Medical Records. Heliyon.

[6] Elvy J., Haremza E., Morris A.J., Whiley M., Gay S. (2023). Blood Culture Quality Assurance: Findings from a RCPAQAP Key Incident Monitoring and Management Systems (KIMMS) Audit of Blood Culture Performance. Pathology.

[7] Shapiro N.I., Wolfe R.E., Wright S.B., Moore R., Bates D.W. (2008). Who Needs a Blood Culture? A Prospectively Derived and Validated Prediction Rule. J. Emerg. Med..

[8] Takeshima T., Yamamoto Y., Noguchi Y., Maki N., Gibo K., Tsugihashi Y., Doi A., Fukuma S., Yamazaki S., Kajii E. (2016). Identifying Patients with Bacteremia in Community-Hospital Emergency Rooms: A Retrospective Cohort Study. PLoS ONE.

[9] Ohno H., Takahashi J., Kato S., Ishii K., Hiramatsu K. (2026). Derivation and Validation of a New Prediction Score for Bacteremia in the Emergency Department. Sci. Rep..

[10] Kiya G.T., Asefa E.T., Abebe G., Mekonnen Z. (2024). Procalcitonin Guided Antibiotic Stewardship. Biomark. Insights.

[11] Becker K.L., Nylén E.S., White J.C., Müller B., Snider R.H. (2004). Clinical Review 167: Procalcitonin and the Calcitonin Gene Family of Peptides in Inflammation, Infection, and Sepsis: A Journey from Calcitonin Back to Its Precursors. J. Clin. Endocrinol. Metab..

[12] Peng K., Zhang X., Li Q., Luo Z. (2025). Presepsin as a Diagnostic Biomarker for Sepsis across Neonates, Children, and Adults: A Meta-Analysis. Biomol. Biomed..

[13] Formenti P., Gotti M., Palmieri F., Pastori S., Roccaforte V., Menozzi A., Galimberti A., Umbrello M., Sabbatini G., Pezzi A. (2024). Presepsin in Critical Illness: Current Knowledge and Future Perspectives. Diagnostics.

[14] Miller J.M., Binnicker M.J., Campbell S., Carroll K.C., Chapin K.C., Gonzalez M.D., Harrington A., Jerris R.C., Kehl S.C., Leal S.M. (2024). Guide to Utilization of the Microbiology Laboratory for Diagnosis of Infectious Diseases: 2024 Update by the Infectious Diseases Society of America (IDSA) and the American Society for Microbiology (ASM). Clin. Infect. Dis..

[15] Palavecino E.L., Campodónico V.L., She R.C. (2024). Laboratory Approaches to Determining Blood Culture Contamination Rates: An ASM Laboratory Practices Subcommittee Report. J. Clin. Microbiol..

[16] Fabre V., Sharara S.L., Salinas A.B., Carroll K.C., Desai S., Cosgrove S.E. (2020). Does This Patient Need Blood Cultures? A Scoping Review of Indications for Blood Cultures in Adult Nonneutropenic Inpatients. Clin. Infect. Dis..

[17] Karlath F.A., Rehan M.A., Geigor A., Mitchell M.J., Arnaout S., Greenough T.C., Ellison R.T. (2025). Retrospective Analysis of the Clinical Significance of Positive Blood Cultures in the Emergency Department: A Single-Center Study. Open Forum Infect. Dis..

[18] Matono T., Yoshida M., Koga H., Akinaga R. (2022). Diagnostic Accuracy of Quick SOFA Score and Inflammatory Biomarkers for Predicting Community-Onset Bacteremia. Sci. Rep..

[19] Singer M., Deutschman C.S., Seymour C.W., Shankar-Hari M., Annane D., Bauer M., Bellomo R., Bernard G.R., Chiche J.-D., Coopersmith C.M. (2016). The Third International Consensus Definitions for Sepsis and Septic Shock (Sepsis-3). JAMA.

[20] Jacobi J. (2022). The Pathophysiology of Sepsis—2021 Update: Part 2, Organ Dysfunction and Assessment. Am. J. Health Syst. Pharm..

[21] Chairaj T., Mongkhon P., Leewongsakorn P., Saensongkwae K., Nangola S., Saoin S., Prompunt E., Chantharit P., Kloypan C. (2025). Diagnostic Performance of Procalcitonin and Presepsin in Sepsis: A Systematic Review and Meta-Analysis. BMC Emerg. Med..

[22] Galardo G., Crisanti L., Gentile A., Cornacchia M., Iatomasi F., Egiddi I., Puscio E., Menichelli D., Pugliese F., Pastori D. (2025). Neutrophil to Lymphocyte Ratio (NLR) and Short-Term Mortality Risk in Elderly Acute Medical Patients Admitted to a University Hospital Emergency Department. Intern. Emerg. Med..

[23] Terradas R., Grau S., Blanch J., Riu M., Saballs P., Castells X., Horcajada J.P., Knobel H. (2012). Eosinophil Count and Neutrophil-Lymphocyte Count Ratio as Prognostic Markers in Patients with Bacteremia: A Retrospective Cohort Study. PLoS ONE.

[24] Zhu N.J., Rawson T.M., Mookerjee S., Price J.R., Davies F., Otter J., Aylin P., Hope R., Gilchrist M., Shersing Y. (2022). Changing Patterns of Bloodstream Infections in the Community and Acute Care across 2 Coronavirus Disease 2019 Epidemic Waves: A Retrospective Analysis Using Data Linkage. Clin. Infect. Dis..

