# Supplementary material for: A Prediction Score Versus Procalcitonin and Presepsin for Predicting Bacteremia in the Emergency Department: A Single-Center Retrospective Cohort Study
Source: Diagnostics (Basel). 2026 Aug 3;16(15):2447. doi: 10.3390/diagnostics16152447 (PMC13465308; doi:10.3390/diagnostics16152447)
Supplement: Supplementary file 1 [file diagnostics-16-02447-s001.zip › diagnostics-4446465-supplementary.pdf]

Supplementary Material

Supplementary Figures

Figure S1. Calibration plots of the prediction score and procalcitonin (Period 1)

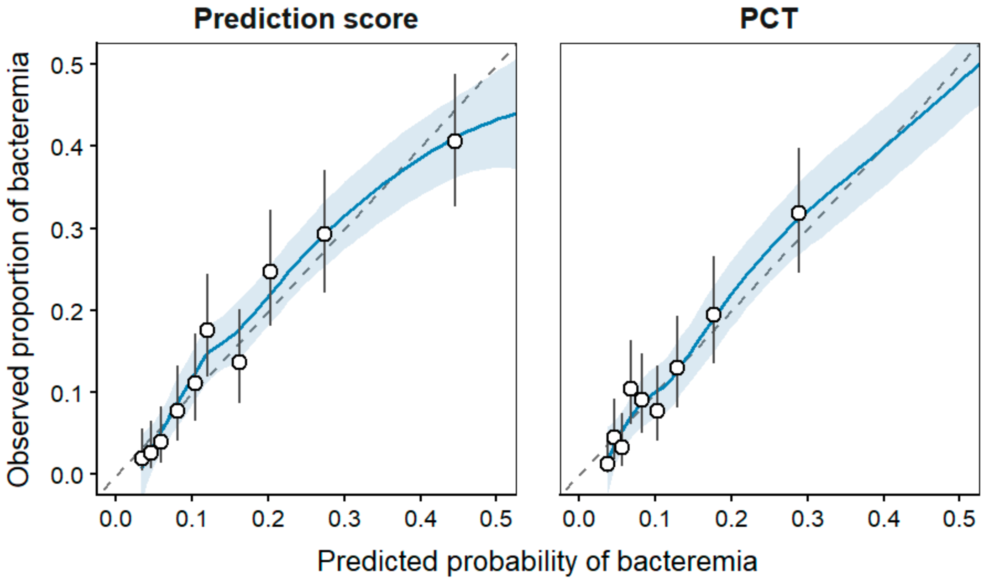

Calibration plots of the prediction score and procalcitonin for predicting bacteremia in Period 1. Circles indicate the observed proportions of bacteremia within deciles of predicted risk, with vertical bars representing their 95% confidence intervals. The blue line and shaded area indicate the loess-smoothed calibration curve and its 95% confidence interval. The dashed line indicates perfect calibration. Abbreviation: PCT, procalcitonin.

Figure S2. Calibration plots of the prediction score and presepsin (Period 2)

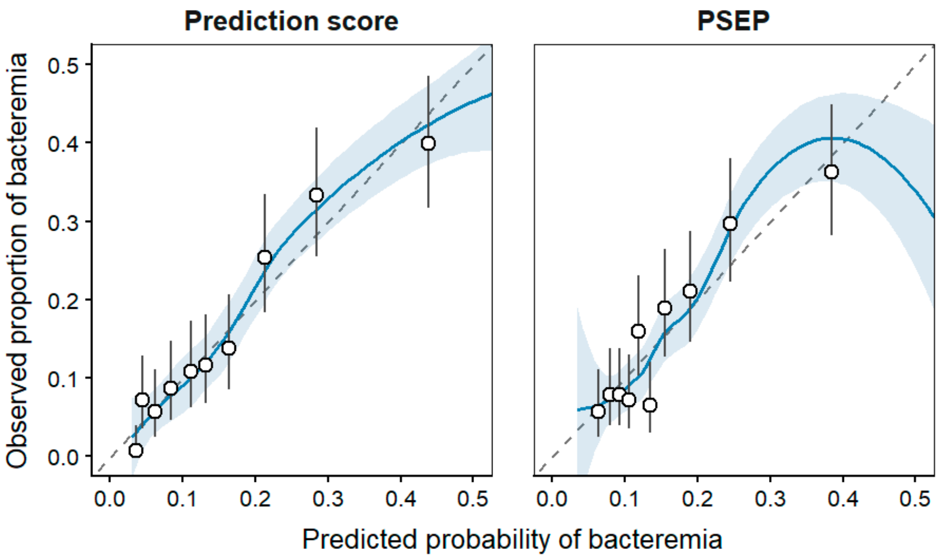

Calibration plots of the prediction score and presepsin for predicting bacteremia in Period 2. Symbols are as described for Figure S1. Abbreviation: PSEP, presepsin.

Supplementary Tables

Table S1. Calibration and overall performance of the prediction score and biomarkers in each period.

Cohort	Model	Optimism-corrected AUC †	Optimism-corrected Calibration slope †	Brier score	Scaled Brier score	Hosmer–Lemeshow p
Period 1	Prediction score	0.76	1.003	0.12	0.11	0.20
	PCT	0.78	0.999	0.11	0.17	0.41

Period 2	Prediction score	0.75	1.002	0.12	0.11	0.24
	PSEP	0.70	1.013	0.12	0.06	0.09

† Optimism-corrected values obtained by bootstrap resampling with 1,000 replications; 95% confidence intervals for the apparent AUC are given in the main text. Calibration intercept was 0 by construction, as models were fitted in each cohort. Abbreviation: PCT, procalcitonin; PSEP, presepsin.

Table S2. Patient screening, exclusion, and inclusion in each period.

	**Period 1 (*n* = 2014)**	**Period 2 (*n* = 1,614)**
**Screened patients**	**2014**	**1614**
**Included patients**	**1539 (76)**	**1383 (86)**
**Excluded patients**	**475 (24)**	**231 (14)**
Ineligible age	42 (2)	51 (3)
Missing data	433 (21)	180 (11)
Procalcitonin	419 (97)	n/a
Presepsin	n/a	167 (93)
Lactate	11 (3)	10 (6)
Body temperature	3 (0.7)	3 (2)

Data are presented as numbers (%). Percentages for screened, excluded, and included patients are calculated against the number of screened patients in each period; percentages for individual missing variables are calculated against the number of patients excluded because of missing data. Patients could have more than one missing variable. Procalcitonin was measured only in Period 1 and presepsin only in Period 2. Abbreviation: n/a, not applicable.

Table S3. Comparison of baseline characteristics, biomarkers, and outcomes between included and excluded patients in each period.

	**Period 1 (*n* = 1972)**			
**Variables**	**Included patients (*n* = 1539 [78%])**	**Excluded patients (*n* = 433 [22%])**	**p value**	**SMD**
Basic characteristics				
Age (years)	75 (63, 84)	74 (56, 83)	0.05	0.12
Male sex	879 (57)	246 (57)	0.95	0.01
Vital signs				
Body temperature (°C)	37.8 (37.0, 38.6)	37.4 (36.8, 38.1)	<0.001	0.31
Laboratory data				
Platelet count (× 10^9/L)	204 (151, 264)	212 (154, 271)	0.15	0.08
Neutrophil lymphocyte ratio	9.1 (5.1, 17.1)	7.7 (4.4, 14.7)	<0.01	0.16
Albumin (g/L)	34 (30, 39)	36 (31, 40)	<0.01	0.18
Bilirubin (mg/dL)	0.8 (0.6, 1.2)	0.8 (0.5, 1.2)	0.62	0.02
Creatinine (mg/dL)	0.8 (0.7, 1.2)	0.8 (0.6, 1.1)	0.05	0.04
Lactate (mg/dL)	13 (10, 20)	13 (10, 19)	0.63	0.02
Procalcitonin (ng/mL)	0.18 (0.06, 0.78)	0.05 (0.04, 0.36)	0.06	0.2
Presepsin (pg/mL)	n/a	n/a	n/a	n/a
Prediction score	4 (2, 6)	3 (1, 5)	<0.001	0.21
Patient condition				
Bacteremia	235 (15)	41 (9)	<0.01	0.18
Sepsis	186 (12)	30 (7)	<0.01	0.18
Septic shock	34 (2)	5 (1)	0.23	0.08
Outcomes				
Hospitalization	1,149 (75)	302 (70)	0.05	0.11
In-hospital mortality	111 (7)	31 (7)	>0.99	<0.01
	**Period 2 (*n* = 1563)**			
**Variables**	**Included patients (*n* = 1383 [88%])**	**Excluded patients (*n* = 180 [12%])**	**p value**	**SMD**
Basic characteristics				
Age (years)	77 (67, 85)	75 (64, 85)	0.37	0.08
Male sex	810 (59)	117 (65)	0.12	0.13
Vital signs				

Body temperature (°C)	37.6 (36.9, 38.4)	37.5 (36.8, 38.2)	0.19	0.06
Laboratory data				
Platelet count (× 10^9/L)	203 (150, 268)	215 (164, 279)	0.20	0.08
Neutrophil lymphocyte ratio	9.8 (5.3, 18.3)	10.2 (5.0, 17.6)	0.78	0.07
Albumin (g/L)	32 (28, 37)	33 (28, 39)	0.24	0.13
Bilirubin (mg/dL)	0.7 (0.5, 1.1)	0.7 (0.5, 1.1)	0.43	0.05
Creatinine (mg/dL)	0.9 (0.7, 1.3)	0.9 (0.7, 1.3)	0.96	0.08
Lactate (mg/dL)	14 (10, 21)	14 (10, 20)	0.22	0.05
Procalcitonin (ng/mL)	n/a	n/a	n/a	n/a
Presepsin (pg/mL)	400 (241, 841)	318 (261, 663)	0.85	0.22
Prediction score	4 (2, 6)	4 (2, 6)	0.45	0.07
Patient condition				
Bacteremia	217 (16)	15 (8)	0.01	0.23
Sepsis	186 (13)	19 (11)	0.34	0.09
Septic shock	45 (3)	5 (3)	0.91	0.03
Outcomes				
Hospitalization	1,110 (80)	144 (80)	>0.99	0.01
In-hospital mortality	149 (11)	20 (11)	>0.99	0.01

Data are presented as median (interquartile range) for continuous variables and numbers (%) for categorical variables. Patients excluded because of age ineligibility were not included in this comparison. Continuous variables were compared using the Mann–Whitney U test and categorical variables using the chi-square test. An SMD greater than 0.1 was considered to indicate a meaningful between-group imbalance. Procalcitonin was measured only in Period 1 and presepsin only in Period 2. Abbreviation: SMD, standardized mean difference; n/a, not applicable.
